# Effects of Zeaxanthin on the Insulin Resistance and Gut Microbiota of High-Fat-Diet-Induced Obese Mice

**DOI:** 10.3390/foods13213388

**Published:** 2024-10-24

**Authors:** Zhibo Jin, Meihong Liu, Hongyu Zhao, Jiahan Xie, Wandi Yin, Mingzhu Zheng, Dan Cai, Huimin Liu, Jingsheng Liu

**Affiliations:** 1 College of Food Science and Engineering, Jilin Agricultural University, Changchun 130118, China; jzb0017@163.com (Z.J.); liumh@jlau.edu.cn (M.L.); jiahan0127@163.com (J.X.); yinwandi0501@163.com (W.Y.); zhengmzhu@163.com (M.Z.); caidan@jlau.edu.cn (D.C.); 2 National Engineering Research Center of Wheat and Corn Deep Processing, Changchun 130118, China; 3 Key Laboratory of TCM Pharmacology, Jilin Academy of Chinese Medicine Sciences, Changchun 130021, China; fixiov5815@126.com

**Keywords:** zeaxanthin, high-fat diet, insulin resistance, PI3K/Akt signaling pathway, gut microbiota

## Abstract

Obesity-induced insulin resistance (IR) can precipitate metabolic disorders such as diabetes. Zeaxanthin, a crucial member of the carotenoid family, has been found to mitigate the damage caused by obesity. However, reports on the effects of zeaxanthin on obesity-induced IR are lacking. Our objective was to examine the metabolic regulatory impacts of zeaxanthin on mice subjected to a high-fat diet (HFD) that triggered IR and to explore their influence on gut microbiota regulation. This study constructed a mouse model of metabolic dysfunction caused by lipid-rich nutritional patterns to investigate physiological and biochemical indices, liver pathway expression, and the intestinal microbiota. The mechanisms by which zeaxanthin improved both IR and glucose metabolic disorders were elucidated. The results demonstrate that zeaxanthin effectively suppressed obesity. The fasting blood glucose, area under curve of oral glucose tolerance test and insulin tolerance test, and homeostatic model assessment–insulin resistance (HOMA-IR) indices in the HFDZEA group decreased by 14.9%, 25.2%, 28.9%, and 29.8%. Additionally, zeaxanthin improved the lipid metabolism and alleviated damage to the liver and pancreas while also activating the PI3K/Akt pathway, regulating hepatic gluconeogenesis and the glycogen metabolism. The number of OTUs in the HFDZEA group increased by 29.04%. Zeaxanthin improved the structure and profile of the gastrointestinal microbiome and enhanced its diversity, increasing probiotics abundance, decreasing pathogen abundance, and thereby ameliorating the dysbiosis of enteric microbial communities in rodents with obesity resulting from excessive fat consumption. The outcomes of our analysis provide a rational basis for advancing zeaxanthin-based nutritional products.

## 1. Introduction

Diabetes, a metabolic disorder, presents a significant risk to human health. The global prevalence of diabetes is continuing to increase, and is expected to reach 7.7% by 2030, impacting almost 439 million individuals [1]. By 2045, it is anticipated that there will be 700 million cases internationally [2]. Diabetes not only impairs human health but also imposes a substantial financial burden, making it one of the most significant public health concerns of the 21st century [3]. Diabetes leads to chronic hyperglycemia and aberrations in the carbohydrate metabolism and consequently gives rise to other diseases such as atherosclerosis, cardiovascular diseases, and ketoacidosis [4,5]. Patients with diabetes typically exhibit insulin resistance (IR), the pathological foundation of diabetes, which is a complex pathological state caused by a reduced insulin sensitivity in insulin target organs due to various factors [6]. Obesity induces the metabolic disorders, signal pathway damage and over-accumulated fat that are the stimuli for IR [7]; the adipose tissue release factors that are involved in the development of insulin resistance and dysbiosis gut microbiota also significantly contribute to IR development [8]. Thus, treating IR and disturbances in glucose and lipid metabolism holds significant importance in delaying and regulating type 2 diabetes mellitus (T2DM). Nowadays, many drugs are utilized clinically for the management of T2DM; however, these drugs often produce side effects and can cause harm to the body [9,10,11,12]. Therefore, developing bioactive compounds that are low cost, safe, and efficient in preventing or improving metabolic diseases has captured the attention of many researchers.

The liver, a primary target organ for insulin, has a vital function in the metabolic processes involved in regulating blood glucose levels (BGL). Obesity-provoked hepatic IR represents a significant factor that contributes to fasting hyperglycemia [13]. The PI3K/Akt cascade represents a quintessential insulin-mediated signaling route; upon insulin binding to its receptor, this pathway is activated, and several key sites within the pathway play important roles in regulating the glucose balance and alleviating IR [14,15]. Insulin receptor activation causes the downstream activation of PI3K and phosphate incorporation into Akt, which then augments GSK3β’s phosphorylation state, decreasing its activity. This promotes the dephosphorylation of glycogen synthase (GS), enhancing glycogen synthesis [16]. Moreover, Akt phosphorylation of downstream FOXO1 inhibits the expression of PEPCK and G6Pase, thereby suppressing gluconeogenesis [17,18]. These mechanisms help to regulate the glucose metabolic balance and are pivotal in the prevention and treatment of hyperglycemia and IR.

The gut microbiota, a complex microbial ecosystem, has an essential function in human health [19]. Research indicates that abnormalities in the gut microbiota are closely associated with the body’s metabolic dysfunctions, serving as a key factor in the genesis and development of metabolic abnormalities. Compared to a healthy state, disorders in glucose and lipid metabolism can have a destructive impact on the gut microbiota, altering its structure and species composition, which leads to pathological changes [20]. The gut microbiota can impact the body through its metabolites. Given the connection between the hepatic portal vein and the mesenteric vein, which are in contact with metabolites and microbes in the digestive tract, the liver’s metabolic and other physiological functions are also influenced by microbial communities. An imbalance between these two can lead to pathological changes in the body [21]. Therefore, enteric microorganisms offer a fresh therapeutic approach to managing IR.

Carotenoids and many other natural compounds have been reported that could improve IR and other metabolic disorders [22,23,24,25]. Zeaxanthin is an important carotenoid widely found in corn, marigolds and other vegetables or fruits [26]. As humans and animals cannot synthesize zeaxanthin on their own, they must obtain it from their diets [27]. Research has demonstrated that zeaxanthin can alleviate oxidative damage, inhibit inflammation, improve cardiovascular disease, halt non-alcoholic fatty liver disease, and prevent complications of diabetes such as diabetic retinopathy [28,29,30,31,32]. Nowadays, zeaxanthin is widely used to treat eye diseases and protect eye health [33,34]. Our previous studies have shown that zeaxanthin can promote the expression of brown and beige adipogenesis markers and mitochondrial biogenesis in 3T3-L1 cells to have an anti-obesity effect, and the intake of 20 mg/kg zeaxanthin can inhibit lipogenesis in mice, promote the browning of white adipose cells, and modulate the gut microbiota, thus playing an anti-obesity role [35,36,37]. Building on our previous study, we found that the insulin sensitivity of mice treated with zeaxanthin was enhanced; therefore, we speculate that obesity can induce IR, and obesity-induced IR may be improved with the intervention of zeaxanthin. And insulin-mediated changes in the signaling pathways and gut microbiota with the intervention of zeaxanthin may alleviate the IR-related phenotype in mice fed with a high-fat diet (HFD). However, the regulatory mechanisms of zeaxanthin on IR provoked by an HFD and its effect on the hepatic glucose metabolism are still unclear, and the underlying mechanism of how it affects insulin resistance via the gut microbiota is yet to be investigated.

Therefore, we devised a rodent model of IR with HFD-triggered obesity to investigate the impacts of zeaxanthin intervention. We assessed various physiological and biochemical markers and utilized molecular biology techniques to analyze their impact on the PI3K/Akt pathway and perform a comprehensive assessment of the intestinal flora. A comprehensive assessment of the intestinal flora was conducted to elucidate the ameliorative actions of zeaxanthin on obesity-induced IR and its regulatory mechanisms, as well as to clarify the correlation between IR and the gut microbiota. The present research strives to offer a new theoretical foundation for the development and application of zeaxanthin as a natural active substance in health food products and for its use in natural therapies for metabolic disorders. Zeaxanthin offers a new research target for regulating intestinal health.

## 2. Materials and Methods

### 2.1. Animal Studies

Four-week-old C57BL/6J mice (Beijing Vital River Experimental Animal Co., Ltd., Beijing, China) were *housed* separately at a controlled temperature (22 ± 2 °C) with a 12 h light-dark cycle and had ad libitum food and water access. Zeaxanthin (Great-Lab Biological, Shenzhen, China) and metformin (Sino-American Shanghai Squibb Pharmaceuticals Ltd., Shanghai, China) were dissolved in 0.5% carboxymethylcellulose sodium (CMC-Na). A week-long adaptation was followed by the random assignment of mice to either the standard diet or *HFD group*. The dietary compositions are provided in Table S1. After four weeks, mice on the HFD were weighed and tested for fasting blood glucose and insulin sensitivity. The mice that demonstrated obesity and IR symptoms were considered to be the successful model. Mice on the standard diet were arbitrarily equally allocated two groups (*n* = 12) to a normal nutrition cohort (STD) gavaged with 0.5% CMC-Na or a zeaxanthin group (STDZEA), which received 20 mg/kg of zeaxanthin via gavage. Similarly, mice on the HFD were equally allocated three groups (*n* = 12) into the HFD group (HFD), which received 0.5% CMC-Na via gavage, the zeaxanthin group (HFDZEA), which received 20 mg/kg of zeaxanthin, and the metformin group (MET), the members of which were given 250 mg/kg of metformin. The dosages of zeaxanthin were ten times the maximum recommended human intake based on equivalent dose, and metformin dosages were determined based on the existing literature [38,39]. Treatments were administered via gavage daily for a total of 18 weeks, with the experiment spanning 22 weeks. The masses and nutritional intake of the mice were monitored daily, with weekly measurements of fasting glucose levels initiated at week 5. The study culminated with a 12 h fasting period for all mice, followed by euthanasia; orbital sinus blood was collected in sterile reservoirs, and cecal contents were also collected. Liver samples were frozen for storage, and tissue samples from the liver and pancreas were conserved in formalin for histopathological examination. The experiment protocol was authorized *by* the Animal Care and Use Committee of the Jilin Academy of Chinese Medicine Sciences (JLSZKYDWLL2019-019).

### 2.2. Biochemical and Histological Analyses

The collected blood was centrifuged to separate the serum (4000 rpm, 10 min, 4 °C). The total cholesterol (TC), triglyceride (TG), high-density lipoprotein (HDL), low-density lipoprotein (LDL), and glycated serum protein (GSP) levels were assessed utilizing commercially available assay kits (Nanjing Jiancheng Bioengineering Institute, Nanjing, China). The serum glucose (GLU), alanine transaminase (ALT), and aspartate transaminase (AST) concentrations were determined as per the standard protocol (Beijing Solarbio Science & Technology, Beijing, China). The serum levels of insulin, leptin, and adiponectin were analyzed using ELISA kits following the standard protocol (Shanghai Enzyme-linked Biotechnology, Shanghai, China). Liver and pancreas tissue samples were fixed in 10% formalin , and then paraffin sections were prepared as per the standard protocol. We stained sections (5 μm) with hematoxylin and eosin (H&E), then the pathological state of the liver and pancreas was determined, and images were obtained.

### 2.3. Oral Glucose Tolerance Test (OGTT) and Insulin Tolerance Test (ITT)

In the 20th week, the mice were fasted for 6 h and then received 2 g/kg of glucose orally. The BGLs were quantified at five time points: 0, 30, 60, 90, and 120 min to generate the OGTT curve. In the 21st week, insulin was injected intraperitoneally at 0.75 U/kg to chart the ITT curve, and BGLs were evaluated at congruent time intervals. The area under the curve (AUC) for both OGTT and ITT was measured via GraphPad Prism 7.0 software (GraphPad Software, La Jolla, CA, USA).

### 2.4. Western Blot

Tissues were homogenized using a high-speed tissue homogenizer pre-cooled with liquid nitrogen. RIPA buffer, supplemented with protease and phosphatase inhibitors, was employed to lyse the tissue samples, and quantitative protein analysis was conducted via a BCA Protein Assay Kit as per the vendor’s protocol (Beyotime Biotechnology, Shanghai, China). After denaturation by boiling for 5 min, equivalent protein quantities underwent SDS-PAGE separation before being transferred to a PVDF membrane. Membranes were blocked with 5% BSA, then incubated overnight with IRS1, p-IRS1, PI3K, Akt, p-Akt, GSK3β, p-GSK3β, GS, p-GS, G6Pase, PEPCK, FOXO1, p-FOXO1 and β-actin primary antibodies at 4 °C and horseradish peroxidase (HRP) secondary antibody. The antibodies were diluted with 3% BSA. The antibody details are presented in the Table S2. The protein bands were visualized using the imaging system through a chemiluminescence method, and densitometric analysis of the bands was conducted by utilizing Image J software (Image J 1.53a) and normalized to β-actin.

### 2.5. Analysis of Mouse Intestinal Microbiota

The intestines of the mice, along with their contents, were harvested and rapidly cryopreserved using liquid nitrogen, and the specimens were maintained at −80 °C until further analysis. Following the protocols, total DNA was isolated from mouse fecal samples by the Fast DNA SPIN Extraction Kit (MP Biomedicals, Santa Ana, CA, USA). The DNA quantification and quality assessment was performed via the NanoDrop platform, and an electrophoretic analysis was conducted on agarose gel to verify DNA integrity. The V3-V4 domain amplification within the 16S rRNA gene was achieved via PCR methodology, with designated forward (ACTCCTACGGGAGGCAGCA) and reverse (GGACTACHVGGGTWTCTAAT) primer pairs. The program of amplification was: (1) initial denaturation at 95 °C for 5 min; (2) 40 cycles of 95 °C for 30 s, 55 °C for 30 s, and 72 °C for 30 s; (3) 72 °C for 7 min; and (4) 12 °C for 10 min and hold at 4 °C. The microbial community analysis was performed utilizing the Quantitative Insights into Microbial Ecology 2 software suite for processing the sequencing output (QIIME 2, version 2019.4) available at [QIIME 2 Documentation] (https://docs.qiime2.org/2019.4/tutorials/, accessed on 3 October 2020). In summary, amplicons were normalized and sequenced by the Illumina MiSeq platform and MiSeq Reagent Kit v3 for paired-end 2 × 300 bp sequencing supplied by Shanghai Personal Biotechnology Co., Ltd. (Shanghai, China). The number of SRA accession is PRJNA1157801.

### 2.6. Statistical Analysis

The experimental outcomes are reported as average values ± SD. Intergroup variations were assessed through one-way ANOVA, with subsequent LSD and Tukey’s tests employed for post hoc comparisons. Comparative analyses between each group were carried out via Student’s *t*-test to estimate significant variances. GraphPad Prism 7.0 software (GraphPad Software, La Jolla, CA, USA) served as the primary tool for our statistical analysis. *p* < 0.05 indicated statistical significance.

## 3. Results

### 3.1. Impacts of Zeaxanthin on the Basic Indices of Mice

**Figure 1:**
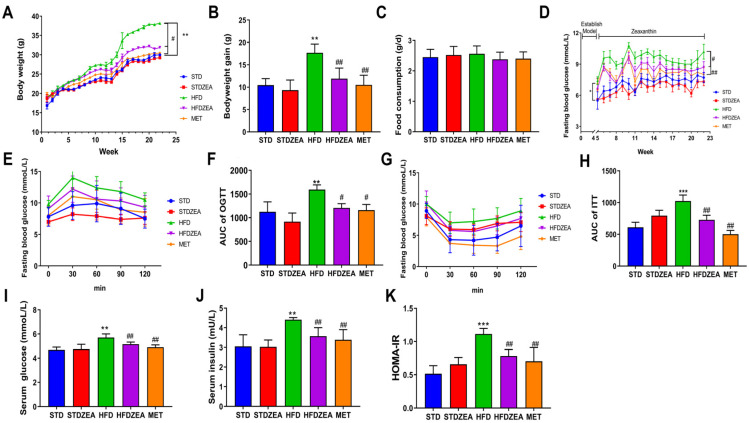
The basic indices of mice. (**A**) Body weight; (**B**) body weight gain; (**C**) food consumption; (**D**) fasting blood glucose; (**E**) OGTT curves (*n* = 8); (**F**) AUC of OGTT (*n* = 8); (**G**) ITT curves (*n* = 8); (**H**) AUC of ITT (*n* = 8); (**I**) serum glucose level; (**J**) serum insulin level; (**K**) HOMA-IR index. Data are presented as means ± SD (*n* = 10)., ** *p* < 0.01, and *** *p* < 0.001 STDZEA, HFD vs. STD; ^#^ *p* < 0.05 and ^##^ *p* < 0.01 HFDZEA, MET vs. HFD.

The initial body weights of all mice were almost identical. After 22 weeks, in contrast to the STD cohort, mice belonging to the HFD group exhibited a significant elevation in body mass, while the HFDZEA group exhibited a significant reduction of 18.4% when compared to the HFD group. The HFDZEA and MET cohorts exhibited comparable body weights, with no significant discrepancies observed (Figure 1A,B). Additionally, the groups exhibited similar feeding behaviors, with no statistically significant differences detected (Figure 1C). Upon conclusion of the modeling phase, the mice maintained on an HFD exhibited significantly increased fasting blood glucose concentrations. After intervention with zeaxanthin, no significant alterations in BGL were detected when comparing the STDZEA group with the STD cohort, indicating that zeaxanthin did not impact BGL in mice on a standard diet. Notably, both HFDZEA and MET groups had significantly lowered BGL compared to the HFD group (Figure 1D), demonstrating that zeaxanthin can improve hyperglycemia in obese insulin-resistant mice.

The results of the OGTT revealed that BGL in the HFD group rose most rapidly, and at 120 min, they remained higher than the baseline. In contrast, the BGL in the HFDZEA group were comparable to the initial values (Figure 1E). As illustrated in the ITT results, the BGLs in the HFD group were raised at all time points compared to the other groups, and by 120 min, the BGL returned approximately to baseline. Following zeaxanthin intervention, the BGL only recovered to 76.23% of the initial levels (Figure 1G). Additionally, compared to the HFD group, the AUC values for the ITT and OGTT in the HFDZEA group were significantly reduced (Figure 1F,H). Regarding insulin, the hormone responsible for mediating BGL balance within the body, the HFD-group mice had significantly higher serum insulin and fasting blood glucose levels than the STD group (Figure 1J), with a significantly elevated homeostatic model assessment–insulin resistance (HOMA-IR) index (Figure 1K). Zeaxanthin treatment significantly reduced these levels. This indicates that zeaxanthin intervention can enhance insulin sensitivity, repair glucose tolerance damage, and has a therapeutic role for IR.

### 3.2. Impacts of Zeaxanthin on Serum Biochemical Parameters

**Figure 2:**
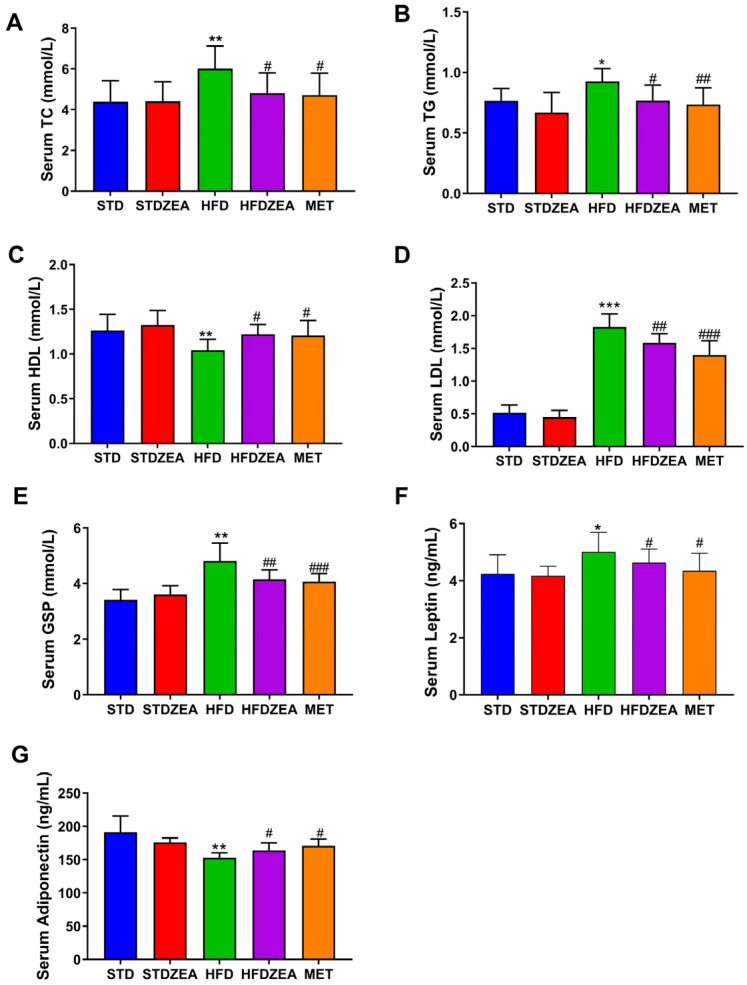
The serum biochemical parameters. (**A**) TC; (**B**) TG; (**C**) HDL; (**D**) LDL; (**E**) GSP; (**F**) leptin; (**G**) adiponectin serum levels. Data are presented as means ± SD (*n* = 8). * *p* < 0.05, ** *p* < 0.01, and *** *p* < 0.001 STDZEA, HFD vs. STD; ^#^ *p* < 0.05, ^##^ *p* < 0.01, and ^###^ *p* < 0.001 HFDZEA, MET vs. HFD.

To further clarify the impacts of zeaxanthin on carbohydrate and fat metabolism in mice consuming an HFD, serum analyses were conducted. The data in Figure 2A–D indicate a significant elevation in TG, TC, and LDL levels, while the HFD group exhibited a significant reduction in serum HDL levels. Zeaxanthin intervention significantly lowered the TG, TC, and LDL serum levels, contrasted with a significant rise in HDL levels, indicating an improvement in the lipid metabolism abnormalities caused by an HFD. Additionally, a significant elevation in GSP levels was exhibited among the HFD group compared to their STD-group counterparts. Zeaxanthin intervention significantly reduced these levels, alleviating the condition of prolonged hyperglycemia (Figure 2E). A significant rise in serum leptin was observed among the mice belonging to the HFD group. Meanwhile, serum leptin levels were significantly reduced in the HFDZEA group, indicating an improvement in leptin resistance (Figure 2F). Notably decreased levels of adiponectin, which enhances insulin sensitivity, were observed in the HFD group compared to their STD counterparts, while the HFDZEA group showed a considerable rebound effect, with adiponectin levels significantly rising to near-typical values (Figure 2G). Overall, zeaxanthin intervention significantly ameliorated lipid metabolism abnormalities in mice rendered obese through high-fat diet intake, relieved abnormalities in glycemic hormonal levels, and led to some improvements in IR.

### 3.3. Impacts of Zeaxanthin on Organ Function

**Figure 3:**
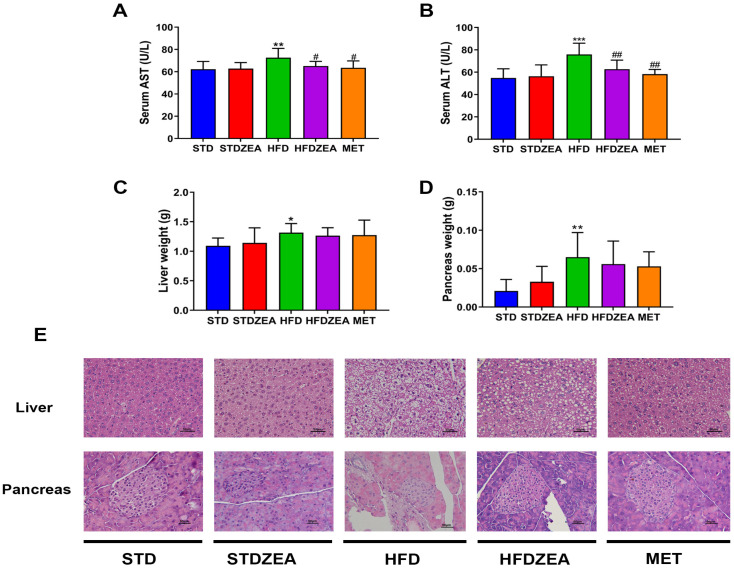
Impact of zeaxanthin intervention on the mice viscera. (**A**) AST; (**B**) ALT serum levels; (**C**) liver weight; (**D**) pancreas weight; (**E**) H&E staining of liver and pancreas (scale bar, 50 μm) (*n* = 5). Data are presented as means ± SD (*n* = 8). * *p* < 0.05, ** *p* < 0.01, and *** *p* < 0.001 STDZEA, HFD vs. STD; ^#^ *p* < 0.05 and ^##^ *p* < 0.01 HFDZEA, MET vs. HFD.

AST and ALT levels are important indicators of liver function. Compared to AST and ALT levels in the STD group, those of the HFD group were significantly elevated (Figure 3A,B). Zeaxanthin intervention induced a significant reduction in AST and ALT, highlighting zeaxanthin’s therapeutic effect on liver function.

Figure 3E shows H&E staining of the liver, which highlights that mice fed a standard diet had well-arranged hepatocytes of normal size with clear and intact structures; however, significant fat accumulation and disarrayed arrangement were evident in the hepatocytes of the HFD group. In the HFDZEA group, hepatocytes tended towards regularity with significantly reduced lipid droplets. Additionally, the liver weight of the HFD group significantly increased, while the zeaxanthin intervention led to a reduction in weight (Figure 3C).

H&E staining of the pancreas (Figure 3E) illustrated that the pancreatic cells of the STD group had clear and complete boundaries with an orderly arrangement. Conversely, the islet cells of the HFD group exhibited a disorganized arrangement and unclear edges, indicating structural damage, which was alleviated after zeaxanthin treatment. Furthermore, an HFD led to a significant elevation in pancreas weight, which was mitigated by the intake of zeaxanthin (Figure 3D).

These findings indicate that zeaxanthin can protect liver function and preserve structural integrity by inhibiting fat accumulation; it also provides some protection to the pancreas.

### 3.4. The Regulatory Effect of Zeaxanthin on Hepatic Glucose Metabolism

**Figure 4:**
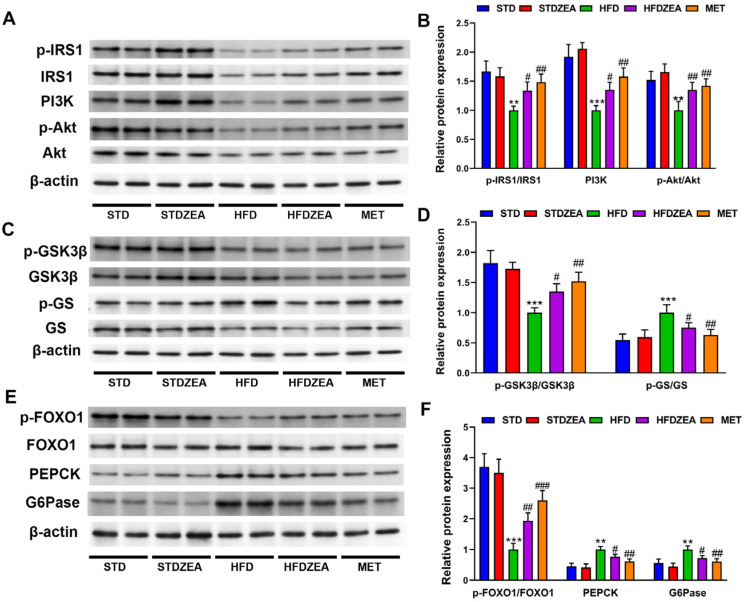
Impact of zeaxanthin intervention on hepatic glucose metabolism and PI3K/Akt pathway protein expression. The protein expression levels of p-IRS1, IRS1, PI3K, p-Akt, and Akt (**A**); p-GSK3β, GSK3β, p-GS, and GS (**C**); p-FOXO1, FOXO1, PEPCK, and G6pase (**E**); the relative protein levels of p-IRS1, IRS1, PI3K, p-Akt, and Akt (**B**); p-GSK3β, GSK3β, p-GS, and GS (**D**); p-FOXO1, FOXO1, PEPCK, and G6pase (**F**) were analyzed via Western blot analysis. Data are presented as means ± SD (*n* = 3). ** *p* < 0.01, and *** *p* < 0.001 STDZEA, HFD vs. STD; ^#^ *p* < 0.05, ^##^ *p* < 0.01, and ^###^ *p* < 0.001 HFDZEA, MET vs. HFD.

Compared to the STD group, the phosphorylation level of the IRS1 (Tyr632) protein in the livers of the HFD group was significantly reduced, while it was significantly increased by zeaxanthin intervention (Figure 4A,B). Additionally, zeaxanthin intervention significantly enhanced the PI3K protein expression and Akt phosphorylation level (Ser473) in obese mouse livers, activating the PI3K/Akt pathway (Figure 4A,B). Once Akt is activated, it can inhibit the activity of the downstream protein GSK3β, thereby promoting the function of GS, a glycogen-synthesizing enzyme. Figure 4C,D shows that the GSK3β protein phosphorylation levels were significantly reduced in the livers of the HFD group compared to the STD group. Conversely, GS protein phosphorylation expression was significantly increased. Zeaxanthin treatment significantly increased the phosphorylation expression of GSK3β in the liver proteins of the obese mice and reduced the GS protein phosphorylation levels, promoting the synthesis of hepatic glycogen.

Akt can also inhibit the activity of FOXO1, thereby reducing the gluconeogenic key factors PEPCK and G6Pase expression. Figure 4E,F shows that our analysis revealed a significant decrease in liver FOXO1 phosphorylation levels among the HFD group compared to their STD counterparts. Nonetheless, the PEPCK and G6Pase protein expression was significantly increased. After zeaxanthin intervention, FOXO1 phosphorylation levels in the obese mouse liver proteins were significantly elevated. Concomitantly, there was a significant decrease in the protein abundance of PEPCK and G6Pase, with their gluconeogenesis being inhibited.

This indicates that zeaxanthin can reduce hepatic gluconeogenesis levels and promote glycogen synthesis via PI3K/Akt signaling, thereby ameliorating the abnormalities in glucose metabolism induced by high-fat-diet-induced IR.

### 3.5. Impact of Zeaxanthin on Gut Microbiota

**Figure 5:**
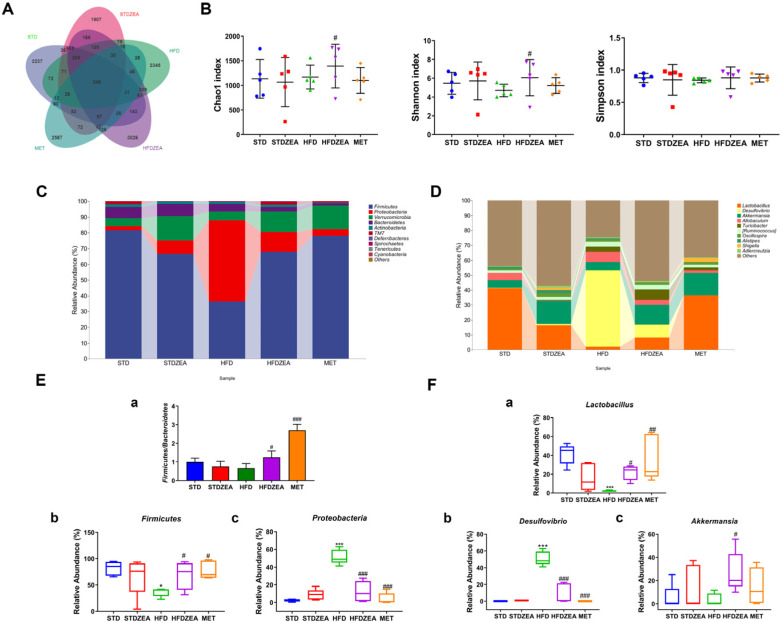
The impact of zeaxanthin on the gut microbiota of mice. (**A**) Venn diagram; (**B**) alpha diversity; (**C**) the relative abundance of top 10 at the bacterial phylum level; (**D**) the relative abundance of top 10 at the bacterial genus level; (**E**) the quantitative relative abundance of the gut microbiota at the bacterial phylum level (a: the *Firmicutes*/*Bacteroidetes* ratio; b: the relative abundance of the *Firmicutes*; c: the relative abundance of the *Proteobacteria*); (**F**) the quantitative relative abundance of the gut microbiota at the bacterial genus level(a: the relative abundance of the *Lactobacillus*; b: the relative abundance of the *Desulfovibrio*; c: the relative abundance of the *Akkermansia*). Data are presented as means ± SD (*n* = 5). * *p* < 0.05, and *** *p* < 0.001 STDZEA, HFD vs. STD; ^#^ *p* < 0.05, ^##^ *p* < 0.01, and ^###^ *p* < 0.001 HFDZEA, MET vs. HFD.

The 16S rRNA gene sequencing was employed to assess the microbiota present in cecal content samples. The rarefaction and abundance curves showed that both the quantity and depth of the samples were adequate for the analysis (Figures S1 and S2). The Venn diagram illustrates that 12,120 operational taxonomic units (OTUs) were recognized across the samples, with each group sharing 248 common OTUs (Figure 5A). Following intervention with zeaxanthin, the number of OTUs unique to the gut microbiota of the HFDZEA group increased to 3026, representing a 29.04% rise in comparison to the HFD group. This suggests that zeaxanthin intervention may enhance the diversity of gut microbiota species.

The alpha diversity indices, which serve as measures of biological diversity, can characterize the richness, diversity, and evenness of species in a specific environment. In rodents that developed adiposity due to consumption of lipid-rich feed , gut microbiota indices, such as Chao1, Simpson, and Shannon indices, all declined. Following the intervention with zeaxanthin, the Chao1 and Shannon indices significantly increased (Figure 5B), indicating that zeaxanthin can improve the extent and heterogeneity of a mouse’s enteric microbiome.

**Figure 6:**
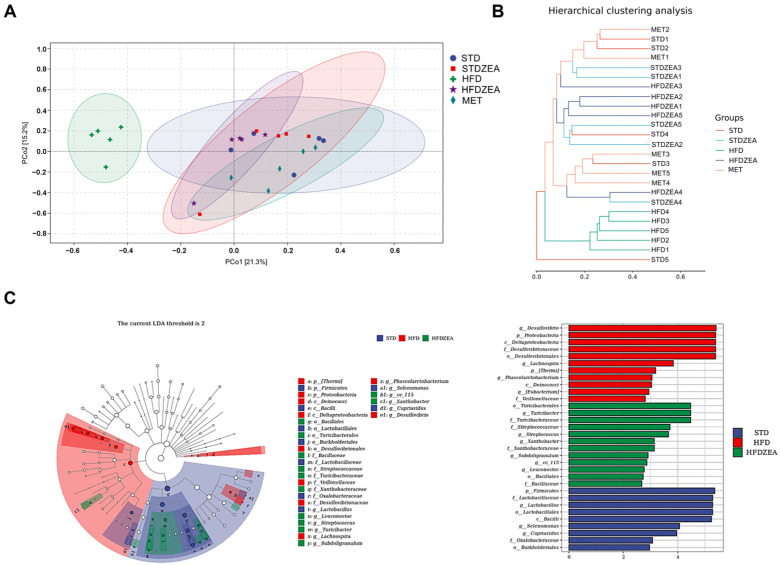
Changes in gut microbiota composition with zeaxanthin treatment. (**A**) Beta diversity and principal component analysis; (**B**) hierarchical clustering analysis; (**C**) LEfSe analysis and LDA analysis. Number of samples (*n* = 5).

Beta diversity assesses the similarities and differences in microbial composition between different samples. In PCoA and hierarchical clustering analysis (Figure 6A,B), the gut microbiota composition of the HFD group was completely separated from the other groups, with no overlap, indicating significant alterations in the murine intestinal microbiome linked to high-fat-diet-induced IR. After intervention with zeaxanthin, the intestinal microbial profile of the mice more resembled that of the control cohort and overlapped, indicating a high similarity and suggesting that zeaxanthin can significantly ameliorate adverse changes in the gut microbiota of IR and obese mice.

The relative abundances at the phylum level (Figure 5C) revealed that the five phyla with the highest relative abundances were *Firmicutes*, *Proteobacteria*, *Verrucomicrobia*, *Bacteroidetes*, and *Actinobacteria*. Intake of a high-fat diet significantly reduced the proportion of *Firmicutes* in intestinal microbial communities while slightly lowering the *Firmicutes*/*Bacteroidetes* ratio. Following zeaxanthin intervention, there was an elevation in the *Firmicutes* abundance in HFDZEA-treated mice, which reverted to STD-like levels, accompanied by a significant elevation in the *Firmicutes*/*Bacteroidetes* ratio (Figure 5E(a,b)). Additionally, as a potential marker for recognizing dysbiosis and disease in the mammalian gut microbiota, *Proteobacteria* flourished under HFD conditions, but this increase was significantly curbed by zeaxanthin or metformin treatment (Figure 5E(c)).

Five major genera dominate the composition of the enteric flora: *Lactobacillus*, *Desulfovibrio*, *Akkermansia*, *Allobaculum*, and *Turicibacter* (Figure 5D). Intake of the HFD caused a significant reduction in *Lactobacillus* which is abundant in probiotics, coupled with an elevation in *Desulfovibrio* which is a pathogen within the murine gastrointestinal microbiome. Zeaxanthin intervention alleviated these trends (Figure 5F(a,b)) and significantly raised the abundance of *Akkermansia* within the intestinal microbial communities of mice who had developed IR due to a lipid-rich diet, indicating that zeaxanthin can modulate the intestinal microbial profile of murine subjects (Figure 5F(c)).

Additionally, this study employed LEfSe (LDA effect size) and LDA (linear discriminant analysis) to analyze the differentially abundant taxa between the HFD and HFDZEA groups. The structural makeup of the gut microorganisms differed significantly across the experimental groups. In the HFD group, the prevalence of *Desulfovibrio*, *Proteobacteria*, and *Deltaproteobacteria* increased. In contrast, in the HFDZEA group, *Turicibacterales*, *Turicibacter*, and *Streptococcaceae* were the dominant taxa (Figure 6C). This indicates that zeaxanthin can counteract dysbiosis by regulating the gut microbiota abundance, thereby maintaining its homeostasis.

### 3.6. Study of the Relation Between Zeaxanthin-Regulated Gut Microbiota and Metabolic Correlations in Mice

**Figure 7:**
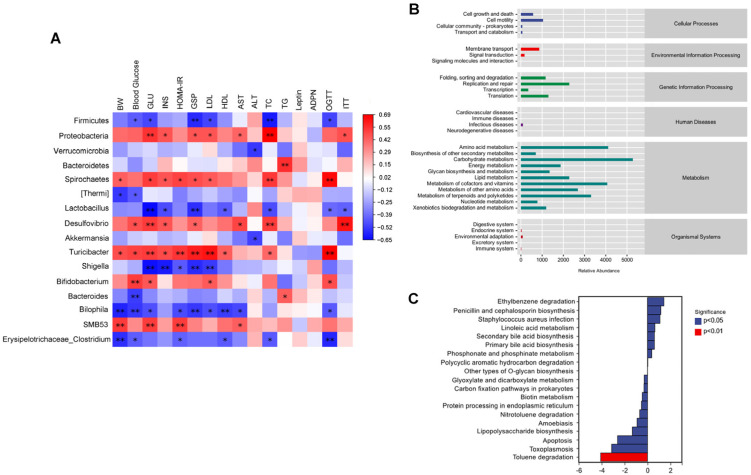
Correlation analysis of the changes in gut microbiota composition caused by zeaxanthin treatment, hepatic glucose metabolism, and physiological indicators. (**A**) Heat map of the relation between gut microbiota and physical–biochemical indicators in various groups (* *p* < 0.05 and ** *p* < 0.01); (**B**) metabolic pathway statistics; (**C**) nineteen KEGG pathways were converted in the HFDZEA group in comparison to the HFD group (*n* = 5).

This study employed Spearman’s correlation analysis to evaluate the interplay between changes in digestive tract microorganisms and indicators associated with IR and metabolic abnormalities. Figure 7A illustrates that *Proteobacteria* and *Desulfovibrio* were positively related to mouse BGL, serum insulin, ALT and TC levels, indicating that an elevation in the abundance of these gut microbiota exacerbates the damage caused by obesity-induced IR. Conversely, *Firmicutes* and *Lactobacillus* were negatively associated with mouse BGL, TC level, and glucose tolerance, suggesting that changes in the abundance of gut microbiota induced by zeaxanthin treatment can improve IR.

Meanwhile, to realize the potential functions and metabolic pathways of the gut microbiome impacted by zeaxanthin, a PICRUSt analysis was performed to predict the metabolic pathways of the gut microbiota (Figure 7B). Through the Kyoto Encyclopedia of Genes and Genomes (KEGG) pathway analysis, functional changes were quantified. Compared to the HFD group, the HFDZEA group exhibited upregulation in nine pathways and downregulation in ten pathways. Metabolic pathways related to glucose metabolism, comprising linoleic acid metabolism and primary and secondary bile acids biosynthesis, were elevated in the HFDZEA group (Figure 7C). This indicates that zeaxanthin can influence metabolic pathways by affecting the gut microbiome, ultimately impacting the glucose metabolism in mice.

## 4. Discussion

Diabetes, characterized by chronic hyperglycemia, is a chronic metabolic disorder that severely threatens bodily health. IR, as a principal symptom and pathological basis of diabetes, also leads to metabolic disorders [4]. Zeaxanthin, a natural compound, has been found to have antioxidant, anti-inflammatory, and anti-obesity qualities [36]. This study reveals that zeaxanthin can improve abnormalities in glucose and lipid metabolism, reduce HOMA-IR, enhance insulin sensitivity, restore glucose tolerance, and regulate carbohydrate metabolism by activating the hepatic PI3K/Akt signaling pathway. Moreover, zeaxanthin regulates the gut microbiome, offering a therapeutic improvement for obesity-induced IR.

Obesity, as a significant contributor to IR, also increases its associated health risks. In this study, an IR model in mice was established using an HFD to induce obesity. The results indicate that zeaxanthin effectively inhibits weight gain and leptin resistance in obese mice, thereby alleviating obesity and offering therapeutic benefits for IR [40]. Obese mice exhibited significantly elevated fasting blood glucose levels, along with a marked increase in GSP levels, indicating a prolonged state of hyperglycemia. This condition not only leads to pancreatic dysfunction but also causes metabolic disorders in the glucose metabolism, resulting in severe IR [41,42]. Additionally, insulin levels were significantly higher in obese mice, indicating a compensatory secretion by the body to maintain metabolic balance. However, due to reduced insulin sensitivity, this did not lead to a reduction in BGL. Moreover, a significant increase in HOMA-IR index in obese mice indicated severe IR. Zeaxanthin caused a pronounced decline in fasting plasma glucose measurements in obese mice and notably decreased both insulin levels and HOMA-IR indices, while also enhancing insulin sensitivity and improving glucose tolerance. It also increased the levels of adiponectin, an insulin-sensitizing agent [43]. Pathological damage to the pancreas was also ameliorated, indicating that zeaxanthin can reduce hyperglycemia and treat obesity-induced IR by enhancing insulin sensitivity.

The liver, the primary organ for energy metabolism, is vital for maintaining metabolic steady-state conditions. Metabolic functions can be affected by prolonged hyperglycemia and thus IR can become disordered [44]. Abnormal glucose metabolism and IR are often associated with dyslipidemia; persistent high glucose levels can result in glucose conversion into fat, thereby causing lipid metabolism abnormalities [45]. Studies have shown that HDL can transport TGs and cholesterol back to the liver from the arteries, where they are broken down and excreted from the body. HFDs may lead to reduced HDL levels [46]. Additionally, raised levels of TC and LDL, alongside lowered HDL levels, can exacerbate glucose metabolic disorders and lead to the development of T2DM, subsequently triggering cardiovascular diseases [47]. Our analysis confirmed a significant reduction in HDL content post-exposure to an HFD. Zeaxanthin intervention effectively suppressed the elevation in serum TG, TC, and LDL levels in obese mice and significantly elevated HDL levels, thereby improving dyslipidemia. When pathological damage occurs in the liver, ALT and AST are released into the serum, and thus their levels can be used as crucial indicators of liver health [48]. The results of our serum analysis showed that in the HFD group there were pronounced spikes in ALT and AST concentrations, whereas zeaxanthin significantly improved this trend. Additionally, the HFD promoted lipid accumulation in the liver, which can further exacerbate IR [49]. H&E-stained liver sections from mice on a high-fat diet demonstrated pronounced vacuolar changes and TG accumulation, which could impact their metabolic function. Intervention with zeaxanthin alleviated these symptoms, suggesting that zeaxanthin can treat metabolic disorders in the liver and repair damage.

The PI3K/Akt pathway, a classic signaling route, is intimately connected to the onset of IR in numerous studies, and activation of this pathway has a critical function in the treatment of IR [50,51]. Insulin, synthesized and secreted via pancreatic β-cells, binds to insulin receptors in target organs such as the liver, causing phosphorylation and activation of the receptor substrate IRS (Tyr632) [52]. PI3K, located downstream of IRS1, has a key function in this pathway. IRS1 activates PI3K, which promotes the phosphorylation of downstream Akt (Ser473), thus activating the pathway [53,54]. The results indicate that in obese mice treated with zeaxanthin , there was a significant elevation in IRS1’s phosphorylation status expression of PI3K. Simultaneously, the phosphorylation status of Akt in hepatic regions intensified, prompting pathway initiation. Akt, as a crucial site within the PI3K/Akt pathway, promotes the downstream GSK3β (Ser9) phosphorylation and GS dephosphorylation, thereby facilitating glycogen synthesis [16]. Studies have also shown that an increase in GSK3β protein expression can affect insulin receptors, inhibiting their expression [55]. Additionally, Akt promotes the phosphorylation of FOXO1, which in turn inhibits PEPCK and G6Pase, suppressing gluconeogenesis. The results show that, in the HFDZEA group, the GSK3β phosphorylation and GS dephosphorylation levels were significantly raised in the liver, and PEPCK and G6Pase expression was notably inhibited. This indicates that zeaxanthin can regulate the glucose metabolic balance by suppressing gluconeogenesis and enhancing glycogen synthesis via PI3K/Akt signaling, thereby decreasing BGL and improving IR.

The gut microbiota, a complex microbial community residing in the gastrointestinal tracts of humans and animals [56,57], has been shown to be related to the enteric bacterial populations and IR in several studies, showing that the microbiota may exert a crucial impact on the progression of metabolic syndromes and represent a potential intervention point for ameliorating IR and its associated metabolic syndrome [58,59,60]. Prolonged hyperglycemia can lead to dysbiosis of the gut microbiota, whereas dietary components can facilitate its reconstruction [61]. Studies indicate that zeaxanthin can enhance the complexity and variety of enteric microorganisms while restoring microbial homeostasis caused by hyperglycemia, normalizing them and leading to an improvement in health status [62]. Zeaxanthin increases the prevalence of *Firmicutes* within the intestinal microbial community, and analyses suggest that this is negatively correlated with BGL and glucose tolerance impairment, aligning with similar findings in some other reports [63,64]. Research has shown that the *Firmicutes*/*Bacteroidetes* ratio is inversely related to BGL and is deemed to be closely linked with improvements in glucose metabolism and IR symptoms. However, zeaxanthin can increase this ratio [58,65]. *Desulfovibrio*, a member of the phylum *Proteobacteria*, produces endotoxins, and its abundance increases with an HFD, correlating positively with BGL and serum insulin levels. The abundance of *Lactobacillus* and *Akkermansia*, probiotics closely linked to glucose homeostasis and carbohydrate metabolism [66,67], may be increased with zeaxanthin treatment. Additionally, *Lactobacillus* is inversely associated with BGL, serum insulin content, and GSP levels. The data demonstrate that an HFD can potentially induce IR and glucose regulation issues by changing the enteric microbiota’s structure and population dynamics, whereas zeaxanthin can adjust the microbial structure and enhance the proliferation of advantageous microorganisms while reducing detrimental bacterial populations, thus restoring glucose metabolic homeostasis and treating IR. Zeaxanthin treatment can upregulate the production of initial and subsequent bile acid compounds, activating Takeda G-protein receptor-5 (TGR-5) via the farnesoid X receptor (FXR), which encourages intestinal L cells to secret GLP-1, thus improving IR and glucose metabolic anomalies [68]. These findings indicate that zeaxanthin can regulate the structure and composition of the gut microbiota, thereby treating IR and glucose metabolic disorders.

In summary, obesity induced by an HFD leads to IR and metabolic disorders. Zeaxanthin intervention can reduce BGL, prompt insulin sensitivity, and improve metabolic abnormalities, thus activating the PI3K/Akt signaling pathway in the liver and regulating glycogen synthesis and gluconeogenesis. Furthermore, zeaxanthin can modify the constitution and organization of intestinal microbial communities, increasing beneficial bacteria and decreasing pathogenic bacteria, and thereby providing therapeutic benefits against IR. To gain a more detailed understanding of how zeaxanthin influences metabolism, researchers could track biochemical and physiological markers over time in the future. In addition, a greater focus can be placed on other primary target organs for insulin and other organelles to explore the effects of zeaxanthin on IR.

## Data Availability

The original contributions presented in the study are included in the article and Supplementary Materials, further inquiries can be directed to the corresponding authors.

## Supplementary Materials

The following supporting information can be downloaded at: https://www.mdpi.com/article/10.3390/foods13213388/s1, Figure S1: Rarefaction curves; Figure S2: Abundance curves; Table S1: Composition and ingredients of animal diets; Table S2: Details of the antibodies.
