# Supplementary material for: Effects of Zeaxanthin on the Insulin Resistance and Gut Microbiota of High-Fat-Diet-Induced Obese Mice
Source: Foods. 2024 Oct 24;13(21):3388. doi: 10.3390/foods13213388 (PMC11544810; doi:10.3390/foods13213388)
Supplement: Supplementary file 1 [file foods-13-03388-s001.zip › foods-3235572-supplementary.pdf]

Supplementary Materials

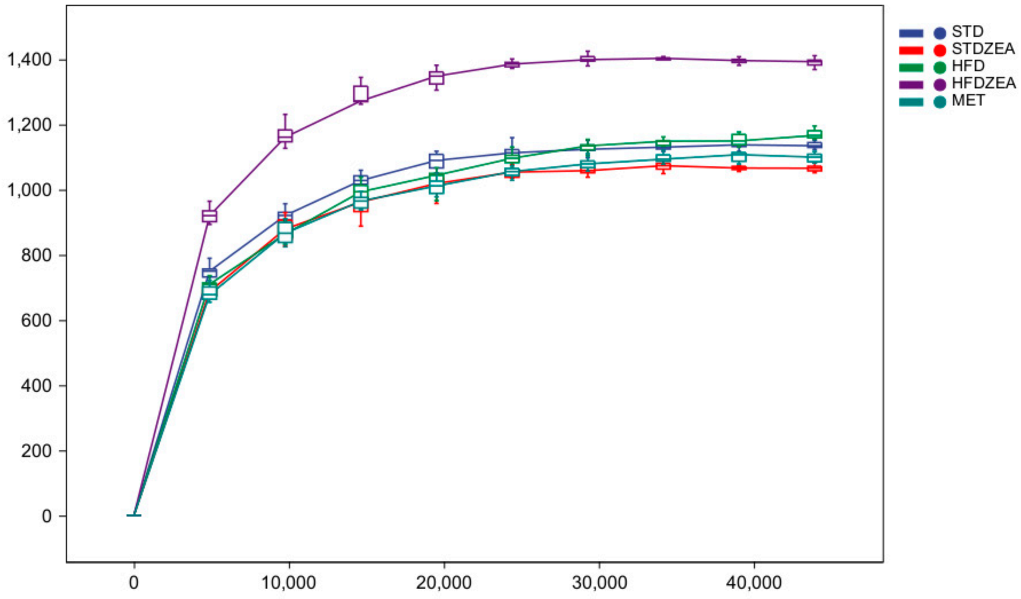

Figure S1. Rarefaction curves.

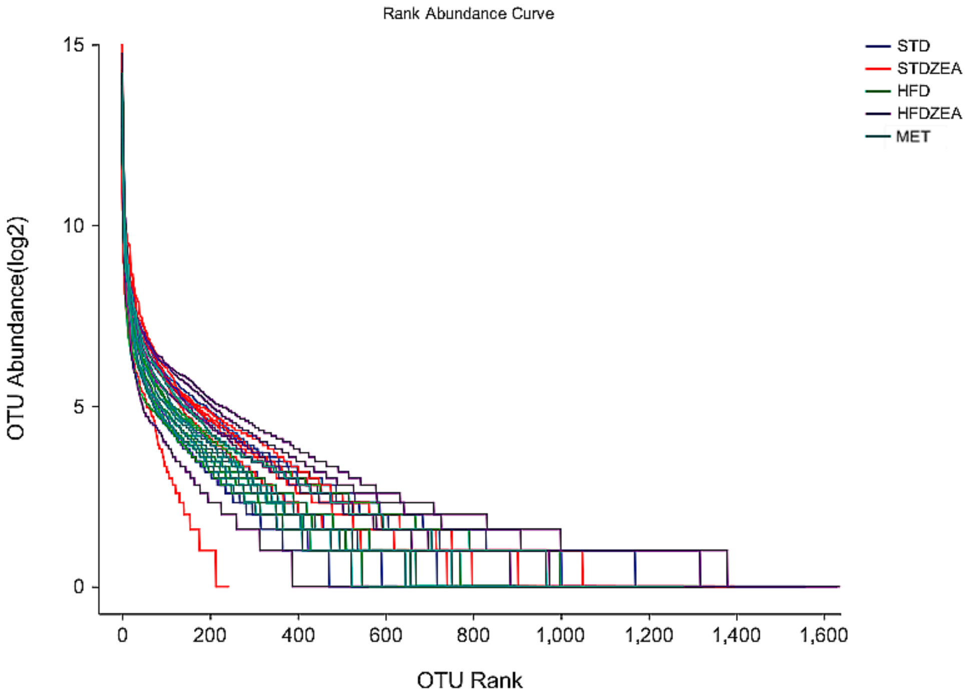

Figure S2. Abundance curves.

Table S1. Compositions and Ingredient of animal diet

	Standard diet		High-fat diet	
Feed ingredient	gm%	kcal%	gm%	kcal%
Protein	19	20	26	20
Carbohydrate	67	70	26	20
Fat	4	10	35	60
Total	--	100	--	100
kcal/gm	3.8		5.2	
Feed ingredient	gm	kcal	gm	kcal
Casein powder (80 mesh)	200	800	200	800
L-Cystine	3	12	3	12
Corn starch	315	1260	--	--
Maltodextrin 10	35	140	125	500
Sucrose	350	1400	68.8	272
Cellulose，BW200	50	--	50	--
Soybean oil	25	225	25	225
Lard	20	180	245	2205
Composite minerals S10026	10	--	10	--
Calcium hydrogen phosphate	13	--	13	--
Calcium carbonate	5.5	--	5.5	--
Potassium Citrate（1H2O）	16.5	--	16.5	--
Multi vitamins V10001	10	40	10	40
Choline bitartrate	2	--	2	2
FD&C Yellow Dye #5	0.05	--	--	--
FD&C Blue Dye #1	--	--	0.05	--
Total	1500.05	4057	773.85	4057

Table S2. The details of the antibodies

Antibodies name	Company& Catalog number
IRS1	ABclonal Cat# A0245
p-IRS1	Bioss Cat# bs-8707R
PI3K	CST Cat# 4249
Akt	ABclonal Cat# A2696
p-Akt	ABclonal Cat# AP1208
GSK3β	ABclonal Cat# A11731
p- GSK3β	ABclonal Cat# AP1088

Continued TableS2.

Antibodies name	Company& Catalog number
GS	ABclonal Cat# A13020
p-GS	CST Cat# 3891
G6Pase	ABclonal Cat# A16234
PEPCK	ABclonal Cat# A4466
FOXO1	ABclonal Cat# A2934
p-FOXO1	ABclonal Cat# AP1379
β-actin	ABclonal Cat# AC038
